# Item

**Published:** 1903-03

**Authors:** 


### ITEM.

Lewis S. Matthews & Co., of St. Louis, Mo., the widely known dealers in medical publications, have removed from 219 North 10th St. to 2623 Olive Street. This house, exclusively devoted to handling medical books and other publications,—American, English, French and German,—offers unusual facilities to purchasers, and solicits correspondence with physicians located in any part of the world.

